# Supplementary material for: Infrared beam-shaping on demand via tailored geometric phase metasurfaces employing the plasmonic phase-change material In3SbTe2
Source: Nat Commun. 2025 Apr 18;16:3698. doi: 10.1038/s41467-025-59122-5 (PMC12008226; doi:10.1038/s41467-025-59122-5)
Supplement: Supplementary file 1 — Supplementary Information [file 41467_2025_59122_MOESM1_ESM.pdf]

Supporting Information for

Infrared Beam-shaping on Demand via Tailored Geometric Phase Metasurfaces employing the Plasmonic Phase-Change Material In_3SbTe_2

*Author(s), and Corresponding Author(s)**

Lukas Conrads[+,,1], Florian Bontke[+,1], Andreas Mathwieser[2], Paul Buske[3], Matthias Wuttig[1], Robert Schmitt[2], Carlo Holly[3,4], Thomas Taubner[#,1]*

+ both authors contributed equally

Affiliations

[1] Institute of Physics (IA), RWTH Aachen University, D-52056 Aachen, Germany

[2] Fraunhofer Institute for Production Technology IPT, 52056 Aachen, Germany

[3] Chair for Technology of Optical Systems, RWTH Aachen University, 52056 Aachen, Germany

[4] Fraunhofer Institute for Laser Technology ILT, 52056 Aachen, Germany

* Email: conrads@physik.rwth-aachen.de

# Email: taubner@physik.rwth-aachen.de

This PDF file includes:

Supplementary Note 1: Dielectric function In_3SbTe_2

Supplementary Note 2: Comparison of IST with other PCMs and usage with active metasurfaces

Supplementary Note 3: Geometric Phase Metasurfaces

Supplementary Note 4: Transmittance spectra IST antennas

Supplementary Note 5: Measured and simulated beam steering metasurfaces

Supplementary Note 6: Metasurface Robustness

Supplementary Note 7: Beam quality after passing the metalens

Supplementary Note 8: Intensity cross-section orbital angular momentum

Supplementary Note 9: Cascaded Metasurfaces

Supplementary Note 10: Diffractive Neural Networks for Dual-hologram design

Supplementary Note 11: Comparison with conventional fabrication techniques

Supplementary Note 12: Measurement Setups

References

Supplementary Note 1: Dielectric Function In_3SbTe_2

The plasmonic phase-change material (PCM) In_3SbTe_2 (IST) features a dielectric amorphous phase and a metallic crystalline phase. The permittivity of IST for the mid-infrared spectral range is shown in **Figure S1**. The permittivity of crystalline IST follows a Drude-like behavior with a negative real part of the permittivity (ε' < 0). The imaginary part (ε'') is zero in the amorphous phase and increases with increasing wavelength for crystalline IST as known from the Drude model. The permittivity values were originally published by Heßler et al.[1] and retrieved by fitting a Tauc-Lorentz-Drude oscillator model to measured infrared spectra. Since the negative real part of the permittivity (ε'<0) directly reveals plasmonic behavior, we chose the permittivity as suitable property to characterize the plasmonic behavior of IST.

Hence, by locally crystallizing IST with precise laser pulses, large-area metasurfaces can be directly programmed within a dielectric surrounding.

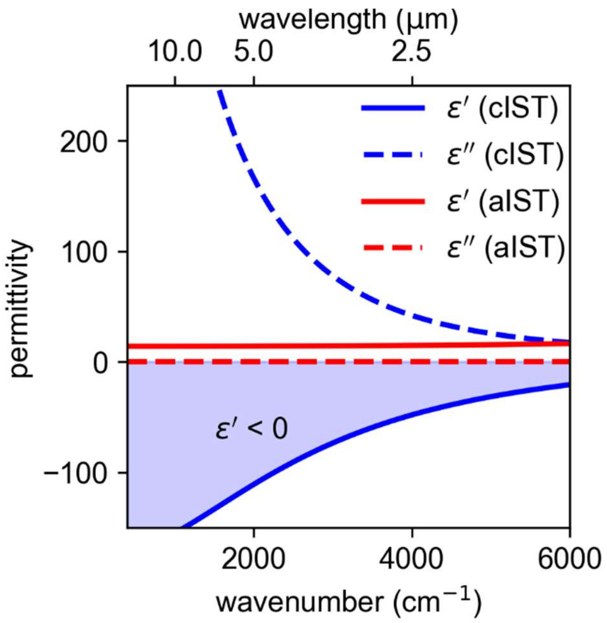

Figure S1: Dielectric function of amorphous (red) and crystalline (blue) IST. The permittivity of crystalline IST follows a Drude-like behavior, while the permittivity of amorphous IST is constant with a value of 14.

Supplementary Note 2: Comparison of IST with other PCMs and usage with active metasurfaces

The plasmonic PCM IST switches from an amorphous dielectric phase to a crystalline metallic one. In the crystalline phase, the permittivity of IST follows a Drude-like behavior (see Supplementary Note 1). Therefore, IST enables direct optical programming of functional metasurfaces by locally crystallizing the PCM with precise laser pulses. This is in strong contrast to conventional dielectric PCMs. Those PCMs are characterized by positive permittivity values in both phases, making dielectric PCMs well suited for metasurface tuning based on a change in the refractive index. **Figure S2** displays the real part (A) and imaginary part (B) of four different materials in the amorphous (solid lines) and crystalline (dashed lines) phases. While conventional dielectric PCMs display an increase of the real part of the permittivity upon crystallization (red curve for $Ge_3Sb_2Te_6$ (GST) and orange curve for $Ge_2Sb_2Se_4Te_1$ (GSST)[2]). The imaginary part for those dielectric PCMs remains nearly zero in both phases. In contrast, the volatile phase-transition material VO_2 and the plasmonic PCM IST feature a negative real part of the permittivity upon crystallization. Therefore, both materials VO_2 and IST show metallic behavior following the Drude-model (also visible in the imaginary part). However, the phase transition of VO_2 is volatile which means that VO_2 remains only metallic if the temperature is above the phase-transition temperature around 68°C. Hence, consistent heating is required to keep the metallic state of VO_2.

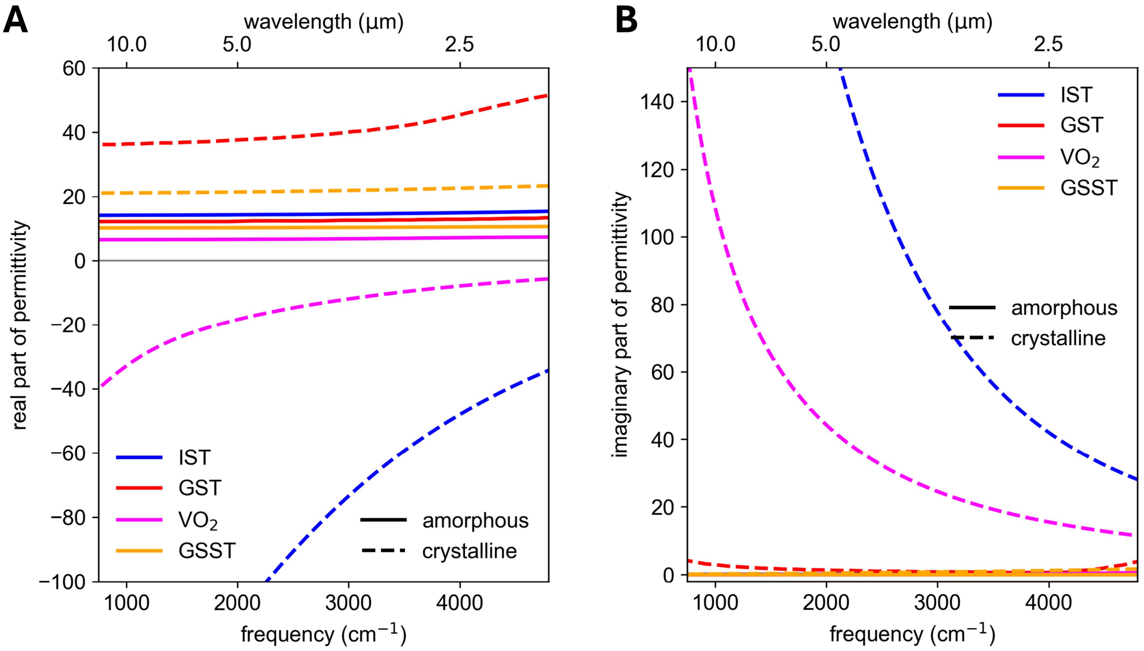

Figure S2: Real part (A) and imaginary part (B) of the permittivity for different PCMs. Dielectric PCMs such as GST and GSST (data taken from ref [2]) feature positive values in the permittivity in both

phases. In contrast, IST and VO_2 undergo a metallic transition and follow a Drude-like behavior in the crystalline phase.

Moreover, we compare our demonstrated work with literature about active metasurfaces to clearly highlight the differences. The results are shown in Table S1.

Table S1: Comparison with active metasurfaces in literature.

reference	metasurface	active material	operation wave-length	fabrication process	switching mechanism	switching time	efficiency
this work	beam steering<br>lensing<br>vortex beams<br>holography	In_3SbTe_2	9 µm	direct programming (single step fabrication)	Laser switching	-	13%
Dong et al.[3]	beam steering<br>vortex beams<br>holography	VO_2	10.6 µm	direct programming (constant heat required)	Laser switching	-	1.2%
Zhang et al.[2]	Integrated photonic switch	$Ge_2Sb_2Se_4Te_1$	1.5 µm	Multiple etching and patterning steps	Electric switching	-	-
Shalaginov et al.[4]	lensing	$Ge_2Sb_2Se_4Te_1$	5.2 µm	Multiple etching and patterning steps	annealing	30 min	24%
Karst et al.[5]	beam steering	PEDOT:PSS	2.65 µm	Etching and patterning	Electric switching	33 ms	40%
Galaretta et al.[6]	beam steering	$Ge_2Sb_2Te_5$	1.5 µm	Lithograpy mask and lift-off	annealing	-	30%
Yin et al.[7]	beam steering<br>lensing	$Ge_3Sb_2Te_6$	3.1 µm	Lithograpy mask and lift-off	annealing	2 min	5-10%
Abdollahramezani et al.[8]	beam steering	$Ge_2Sb_2Te_5$	1.5 µm	Lithograpy mask and lift-off	Electrical switching	200 ns – 200 µs	80%

All previously demonstrated concepts in literature require either multiple mask patterning and etching steps[6–8], or providing a constant temperature to omit switching back in the original state[3]. While these examples mostly demonstrate switchable functionalities by incorporating active materials, our concept showcases fast and flexible design and fabrication of metasurfaces with different functionalities.

The efficiency of our metasurfaces is comparable to other works employing plasmonic geometric phase metasurfaces and is limited mainly by the polarization conversion. Cascading different metasurfaces as demonstrated in Figure 5 in the main manuscript increases the efficiency by further converting the remaining incident polarization. The efficiency could be also enhanced by increasing the antenna density to establish smoother phase gradients, or employ sophisticated antenna designs and layerstacks to suppress reflection and minimize transmitted incident chirality.[9,10]

The multitude of different beam-shaping applications shown in our manuscript excels all previous works mostly focusing on a single functionality.

Supplementary Note 3: Geometric Phase Metasurfaces

Exploiting rotated metallic antennas in combination with circularly polarized light provides a convenient way in tailoring the phase of the employed metasurfaces at will.

In particular, the transmitted light for incident left-handed circularly polarized (LCP) light E_T^L through such a metasurface consisting of anisotropic scatterers, e.g. rod antennas, is given by:[11]

$$E_T^L = \frac{t_o+t_e}{2} E_I^L + \frac{t_o-t_e}{2} \exp(im2\beta)\, E_I^R \quad (1)$$

Here, the t_o and t_e refer to the scattering coefficients for incident linearly polarized light along the two principal axes, and m describes the handedness of the circular polarization, being ±1. Hence, the transmitted light features a component with the same handedness of the incident polarization E_I^L and a component with opposite handedness, i.e. right-handed circularly polarized (RCP) light E_I^R with an additional phase of m2β, also called Pancharatnam-Berry Phase. The phase is only controlled by the rotation of the antenna and the chirality of the scattered light is reversed with respect to the incident light, allowing for clear distinction of the incident light from the scattered one. In particular, the phase ϕ of the scattered light depends on the rotation angle β via:

$$\phi = 2 \cdot \beta \quad (2)$$

A visualization of this concept is shown in Figure S3.

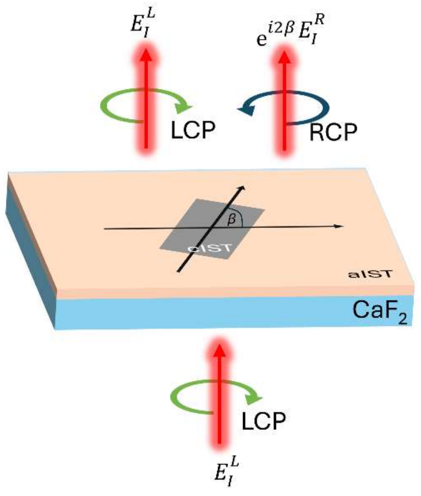

Figure S3: Concept of geometric phase metasurface. The transmitted light features a component with the same handedness of the incident polarization and a component with opposite chirality and an additional phase dependent on the rotation angle of the antenna.

Supplementary Note 4: Transmittance spectra IST antennas

The measured transmittance spectra of crystalline IST rod antennas with varied antenna length with light polarized parallel to the long antenna axis are shown in **Figure S4**. A broad minimum in the transmittance corresponding to the electric dipole resonance of the rod antennas occurs. The broad antenna resonances are beneficial for broadband operation, not limiting the operation wavelength of the metasurface which conventionally is the case for narrow dielectric resonators. For increased antenna length, the electric dipole resonance shifts towards larger wavelengths. Antenna arrays with a length of 2.5 µm display an electric dipole resonance at 9.1 µm and are therefore used for the different metasurfaces. The results are in good agreement with previous experiments about the resonance position of crystalline IST antennas.[1]

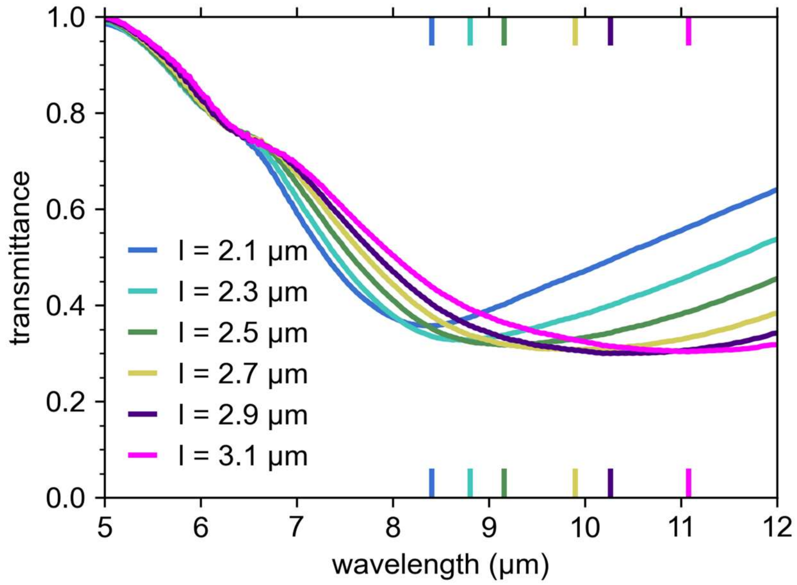

Figure S4: Measured FTIR transmittance spectra of crystalline IST antennas with varied antenna lengths.

Supplementary Note 5: Measured and simulated beam steering metasurfaces

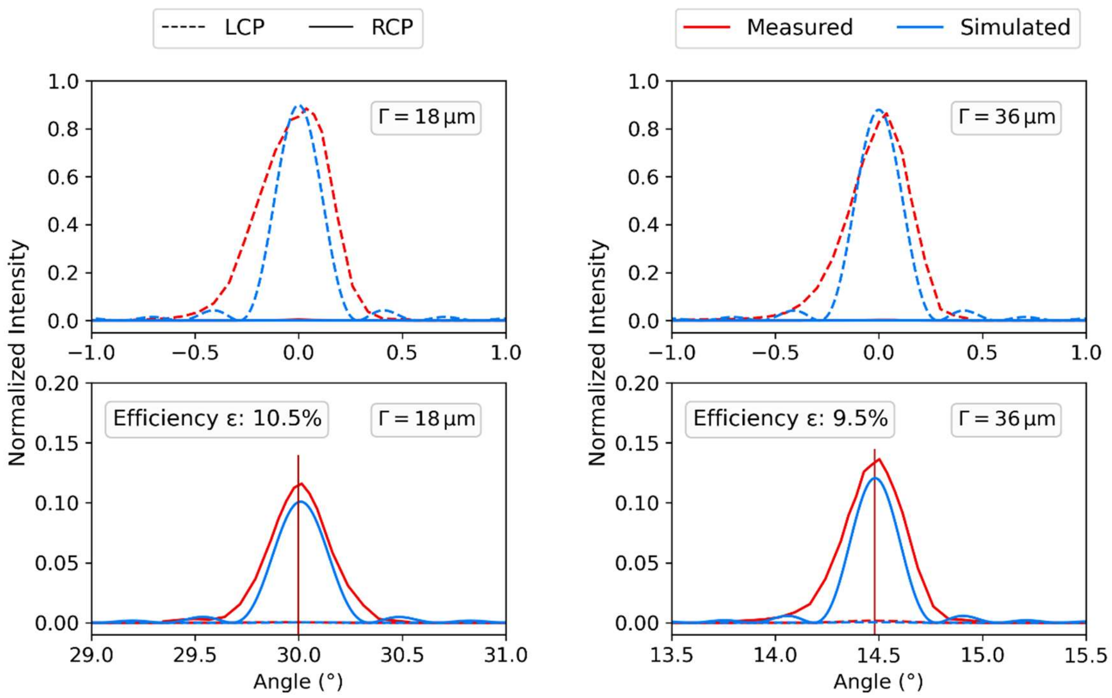

Figure S5: Measured and simulated beam intensities after passing the two beam steering metasurfaces.

Supplementary Note 6: Metasurface Robustness

Commonly, metasurface fabrication requires high precision and accuracy of the fabricated nanoantennas without significantly decreasing the metasurface performance. Since the electric dipole resonances of the employed IST antennas are very broad at 9 µm with a full-width-half-maximum of 4.4 µm, we demonstrate robustness against fabrication imperfections indicating broad band performance of our metasurfaces. Therefore, we fabricate three different beam steering metasurfaces with a supercell period of 18 µm and modified antenna lengths varied from 2.2 µm to 2.9 µm. Light microscope images of the supercells for the different metasurfaces are shown in **Figure S6A**. The corresponding measured transmittance spectra of antenna arrays with varied lengths (see **Figure S6B**) display broad electric dipole resonances at 8 µm for antennas with a length of 2.2 µm shifted to 10.5 µm for antennas with a length of 2.9 µm. Since the applied quarter-wave plates limit the operation wavelength of the metasurfaces to 9 µm, we compare the beam deflection efficiencies for varied antenna lengths as displayed in **Figure S6C**. Because we keep the supercell period constant, the deflection angle is fixed to 30°. The general performance of the metasurfaces feature normalized beam deflection efficiencies around 10 %, despite of the strongly differing resonance positions. Moreover, the deflection intensities of the beam steerer with largest antenna lengths exceeds the optimum case with respect to the resonance position at the operation wavelength. This behavior is further investigated by numerical far-field simulations of the scattered electric field in the xz-cross-section for single antennas (c.f. **Figure S6D**). Here, the lower hemisphere corresponds to scattered light in the substrate closely related to the transmitted light. Remarkably, the antennas with a length of 2.9 µm exhibit more pronounced scattering in the forward direction (lower hemisphere) instead of the backward direction (upper hemisphere) compared to smaller antennas. This explains the difference in the measured beam deflection intensities for varied antenna lengths.

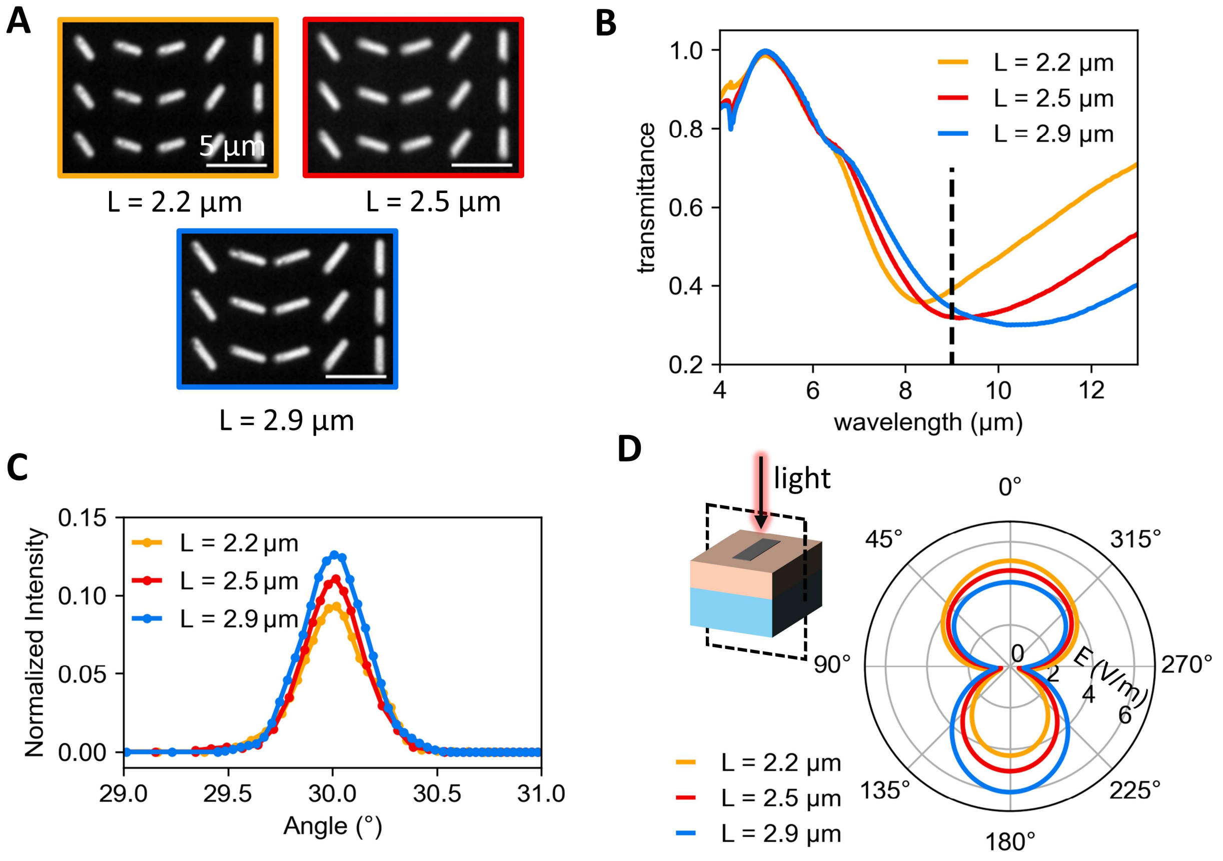

Figure S6: Beam steering performance for varied antenna length. A) Light microscope images of three different beam steering metasurfaces with varied antenna lengths from 2.2 µm to 2.9 µm. **B)** Measured transmittance spectra of antenna arrays with the three different antenna lengths with electric dipole resonances from 8 to 10.5 µm. The black dashed line indicates the operation wavelength of 9 µm. **C)** Measured beam deflection intensities for the different metasurfaces consisting of 3 different antenna lengths. **D)** Simulated far-field electric field scattering in the xz-cross-section (see inset) showing enhanced field scattering for the 2.9 µm long antenna.

Supplementary Note 7: Beam quality after passing the metalens

The measured beam waist of the infrared laser after passing the metalens is shown in **Figure S7**. We fitted the evolving beam width with the formula $w(z) = w_0\sqrt{1+\left(\frac{z}{z_R}\right)^2}$ to determine the beam quality $M^2 = \pi\frac{w_0^2}{z_R}$ after the metasurface. Here, z_R refers to the Rayleigh length and w_0 refers to the beam waist in the focal spot. From the fit, we obtain a beam quality M^2 of 0.97 ± 0.03, which is very close to the optimal case of $M^2 = 1$. Therefore, we conclude that our metasurface even improves the intrinsic beam properties (see **Figure S8**). The unphysical value of M^2 smaller than one might be caused by imprecision during the measurements.

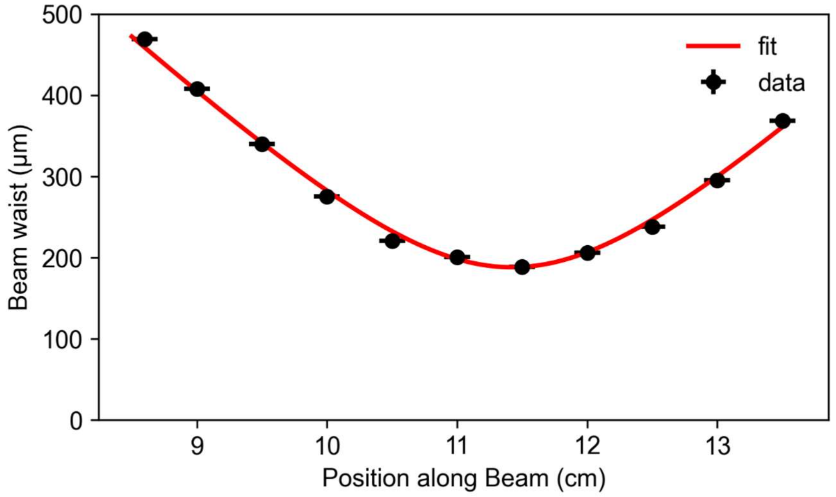

Figure S7: Beam waist after passing the metalens.

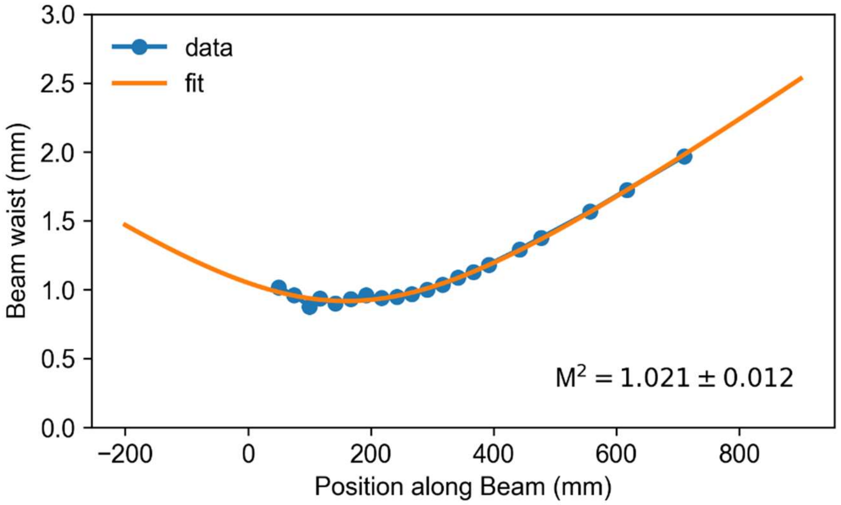

Figure S8: Initial beam characteristic exhibiting an M^2 value of 1.021.

Supplementary Note 8: Intensity cross-section orbital angular momentum

We measured the intensity of the shaped laser beam after passing the orbital angular momentum (OAM) metasurfaces with the knife-edge method to determine the diameter of the observed ring-like intensity patterns (see **Figure S9**). For the metasurface with encoded OAM of l = 1, the ring exhibits a diameter of 1.8 mm, while the ring obtained with the metasurface of encoded OAM of l = 3 features a diameter of 2.6 mm and for l = 5, the ring has a diameter of 3.4 mm.

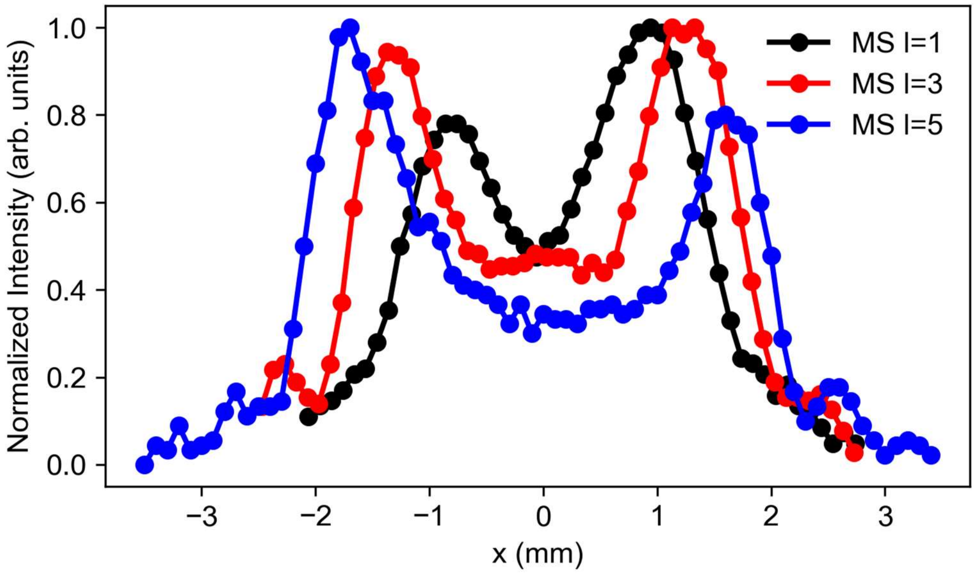

Figure S9: Intensity cross-section of laser beam after OAM metasurface at a distance of 15 cm.

Supplementary Note 9: Cascaded Metasurfaces

As already mentioned previously, the conversion efficiency of our applied metasurfaces is in the range of 10% (c.f. Figure 2 in the main manuscript and Supplementary Note 2). Hence, it is possible to cascade different metasurfaces and take advantage of the non-affected incident polarization of the light after passing through the first metasurface. This concept is visualized in **Figure S10A**. The first metasurface leads to a hologram as already demonstrated in Figure 4 in the main text. Afterwards, the incident LCP polarized light passes the first metasurface and hits the second metasurface designed for a second hologram which will be deflected due to the superimposed phase profile of a beam steerer. The incident LCP light is filtered out. The resulting measurement on the screen is shown in **Figure S10B**. At 0°, the first hologram is visible. Adjacent at 10° deflection angle, the second 'ir nano' hologram appears. Note that the order of the metasurfaces can be interchanged without affecting the result.

Therefore, the unaffected incident polarized light can be further used by multiple metasurfaces for different functionalities.

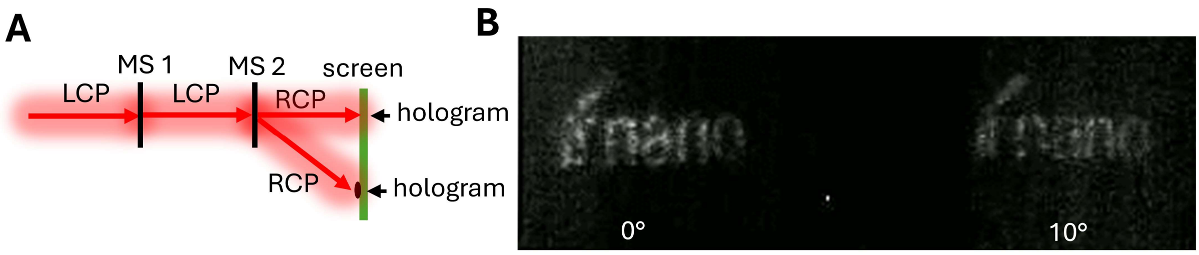

Figure S10: Cascaded metasurfaces. A) Schematic sketch of measuring two cascaded metasurfaces. The first metasurface (MS1) is designed to create a hologram. The non-affected incident LCP light is further directed to the second metasurface (MS2) designed for a second hologram deflected by 10°. **B)** Measurement of the screen showing the first hologram at 0° and the deflected hologram at 10°.

Supplementary Note 10: Diffractive Neural Networks for Dual-hologram design

We design the phase mask of the dual-hologram by treating it as a single layer diffractive neural network[12], which is a stochastic gradient descent approach. This approach utilizes the analytical gradients provided in machine learning computation infrastructures by treating phase masks as trainable layers. This means that the entire optical system can be conceptualized as a physical neural network, with the input beam's complex amplitude as the input layer, the metasurface as the (here single) trainable layer and two output layers. The phase value of each pixel acts as an equivalent to a neuron in conventional neural networks. The connections between the layers are realized through Huygens' principle, so each "neuron" is connected to all previous and following nodes through spherical wave propagation. After implementing all the optics, the training can be performed exactly as a conventional neural network in an arbitrary neural network training environment - in this case in Pytorch Lightning. More details about the method are provided in ref[12] and ref[13]. We choose two separate target planes with two different target intensity distributions at distances of 16 cm and 21 cm respectively, measuring from the position of the metasurface plane. As the target distributions are significantly larger than the metasurface, the second distribution is scaled in its spatial extension to approximately fit to the divergence resulting from expanding the incoming beam to the size of the first target distribution.

Additionally, we employ two regularization techniques[14]. First, weight decay is used to penalize excessively large phase weights. Second, Laplacian regularization minimizes the second derivative between adjacent pixels. This encourages continuous and smooth phase masks, facilitating manufacturing and reducing speckle noise in the calculated intensity distributions.

The Laplacian regularization is implemented as a convolution with a Laplacian kernel

$$K = \begin{matrix} 0 & 1 & 0 \\ 1 & -4 & 1 \\ 0 & 1 & 0 \end{matrix}$$

so that the second derivative of the phase mask ϕ_{mask} can be expressed as

$$\Delta\phi_{\text{mask}} = K \cdot \phi_{\text{mask}}$$

with the Laplace operator Δ. Then, the total loss function is given as

$$L_{total} = \sum_{n=0}^{1} \left\| I_n - I_n^{Target} \right\|^2 + \lambda_1 \|\phi_{mask}\|^2 + \lambda_2 \|\Delta\phi_{mask}\|^2$$

with $I_{0,1}$ as the respective two intensity distribution predictions and λ_i as regularization hyperparameters.

The target intensity distributions displaying 'aIST' and 'cIST' with different lattice structures at the different image planes are shown in **Figure S11A**. The simulated far-field intensity distributions of the obtained metasurface phase mask are shown in **Figure S11B**, revealing good agreement with the target designs.

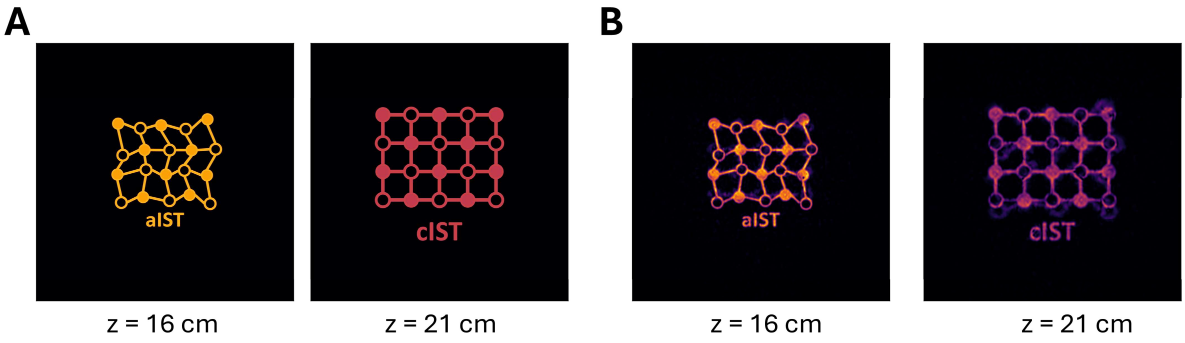

Figure S11: Targeted (A) and simulated far-field intensity distribution of the dual-hologram metasurface.

Supplementary Note 11: Comparison with conventional fabrication techniques

Conventionally, the fabrication of optical metasurfaces is a complex and time-consuming process. The schematic principle of a fabrication process via electron beam lithography or laser lithography is displayed in **Figure S12A**. The substrate material is covered with a polymer resist (i) for example via spin-coating. Afterwards, electrons or a laser modifies the resist (ii), which is subsequently removed with a developer (iii) to create the targeted mask of the structures. In a next step, the target material is deposited onto the previously obtained mask (iv). Finally, the remaining resist also covered with the target material is removed in a lift-off process (v). The fabrication of more complex structures requires even multiple repetitions and aligning steps of the previously described procedure.[15,16]

Even more cumbersome is deep ultraviolet lithography, involving multiple etching steps to finally achieve the targeted nanostructures (see **Figure S12B**).[16]

The overall time to fabricate metasurfaces with the previously discussed approaches is estimated as follows: several hours for patterning the sample with the electron beam or laser, 30 minutes to 1 hour for the development, 1-2 hours for the metal deposition and subsequent 30 minutes for the lift-off process. Each additional fabrication step increases the allocated time for fabrication. If now the availabilities of the different machines are taken into account, up to several days are often required until the metasurface is fabricated.

In contrast, our approach of direct optically programming functional metasurfaces is much simpler (c.f. **Figure S12C**). Here, a thin layer of amorphous IST is deposited onto the substrate (ii) and subsequently crystallized via laser irradiation (iii), leading to plasmonic nanostructures directly written into a dielectric surrounding. Moreover, employing IST allows for post-fabrication adaptations of once written nanoantennas by locally addressing the antenna ends with precise laser pulses.[1,17–19] This is not possible for conventional fabrication techniques where the size and shape of metallic or dielectric nanoantennas are fixed after fabrication.

In summary, our proposed concept speeds up fabrication and prototyping of metasurfaces by omitting time-consuming etching and developing steps. While the required energy to crystallize IST is comparable to other direct laser writing techniques for patterning resist

masks in the range of several 10 mW, significant energy and costs can be saved by omitting the subsequent processing steps.

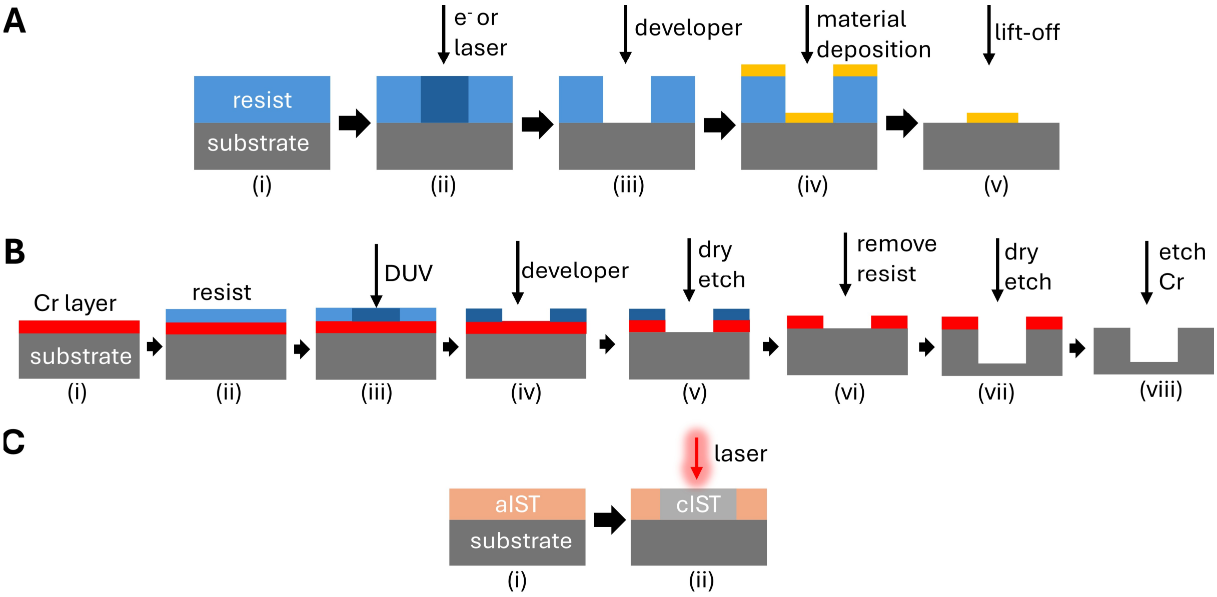

Figure S12: Comparison of different fabrication techniques of metasurfaces. A) Conventional metasurface fabrication such as electron beam lithography involves the deposition of a resist mask (i) with subsequent patterning the resist via electrons (e-) or with a laser (ii). Afterwards, the resist developed (iii), leading to a positive or negative mask. After deposition of the target material (iv), the remaining resist with the material is removed with a lift-off or etching process (v). Complex structures involve multiple repetitions of the described procedure. **B)** Deep ultraviolet lithography involves even more steps, including chromium deposition (i), resist deposition (ii), irradiation of the resist (iii), developing the modified resist (iv), dry etching chromium (v), removing the remaining resist (vi), dry etching the substrate (vii) and finally removing the remaining chromium (viii). **C)** Our approach of fabricating functional metasurfaces involves only the deposition of an amorphous IST layer (i) with subsequent direct optical programming of the plasmonic crystalline IST antennas (ii).

Supplementary Note 12: Measurement Setups

For characterization of the different fabricated metasurfaces, we employed different measurement setups (see **Figure S13**). For all setups, a quantum cascade laser from Daylight Solution with an operation wavelength of 9 µm is attenuated with a variable step attenuator (VA) and then directed to a quarter wave plate (QWP) to create left-handed circularly polarized (LCP) light. After transmitting the metasurface, the light features two components: Partially, the light is scattered by the metasurface and the handedness of the polarization is transformed, i.e. RCP light (as already explained in Supplementary Note 3), and the other component describes the incident LCP light which has not interacted with the metasurface. The incident LCP light is then filtered out with a second QWP and a rotated linear polarizer (LP).

For the beam steering metasurfaces (Figure 1 in main text), the detector is moved along a semicircle to detect the deflected RCP light. A QWP and a linear polarizer (LP) enables a clear distinction between both polarization chiralities (c.f. Figure S13A). In another experiment, the intensity profile of the metalens (Figure 2 in main text) is investigated. Therefore, the knife-edge method is used to determine the beam intensity profile at different positions behind the metalens measured with power sensor after filtering the incident LCP light (c.f Figure S13B). Accordingly, a razor blade is mounted at a micrometer stage and the varied laser power dependent on the position of the razor blade within the beam is recorded. The discrete derivative of the recorded power yields the lateral beam profile. The hologram and the OAM metasurfaces (Figure 3 and 4 in main text) are directly imaged with a thermal camera (TC) on a screen (c.f. Figure S13C and D). The spiral intensity pattern of the OAM metasurface caused by the direct interference with the incident light is obtained by slightly rotating the linear polarizer in order to achieve approximately similar laser powers of the converted RCP and incident LCP light. Finally, the dual-hologram (Figure 6 in main text) is directly imaged with a laser profiling camera Pyrocam IV due to increased sensitivity and more camera pixels to resolve the fine details of the targeted structure (c.f. Figure S13E).

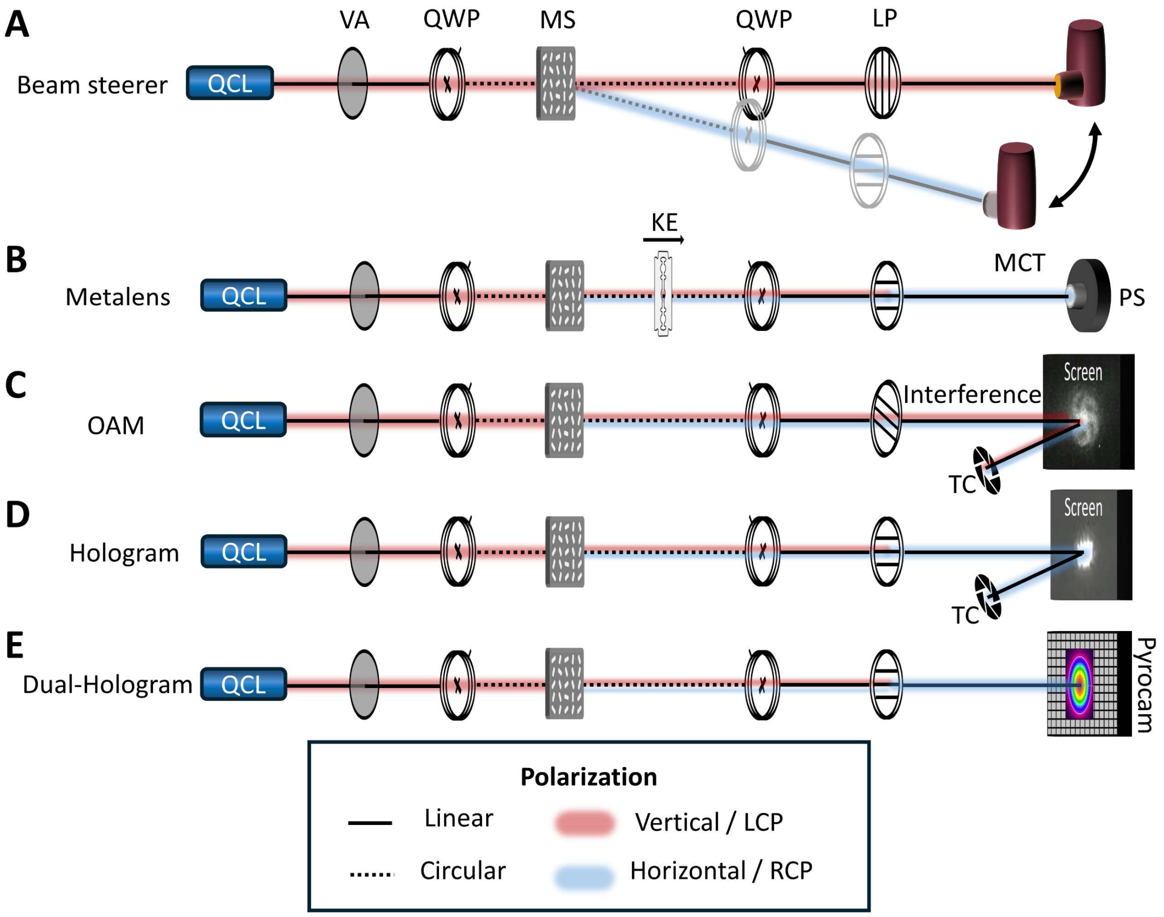

Figure S13: Measurement Setups. A) Characterization of the beam steering metasurfaces with angle-resolved measurements by rotating a detector on a semicircle. **B)** Characterization of the metalens by moving a knife-edge razor blade through the beam path and detecting the intensity with a power sensor (PS). **C)** The OAM metasurfaces are imaged at a screen with a conventional thermal camera (TC). Direct interference is obtained by rotating the linear polarizer (LP) to create equal intensity distributions of the incident light and the scattered light by the metasurface. **D)** The hologram is imaged with the TC on a screen. **E)** For improved accuracy and more detection pixels, the dual-hologram is directly imaged with a Pyrocam after filtering the incident LCP polarization at various distances behind the metasurface.

References

1. Heßler, A. *et al.* In3SbTe2 as a programmable nanophotonics material platform for the infrared. *Nature Communications* **12,** 924; 10.1038/s41467-021-21175-7 (2021).
2. Zhang, Y. *et al.* Broadband transparent optical phase change materials for high-performance nonvolatile photonics. *Nature Communications* **10,** 4279; 10.1038/s41467-019-12196-4 (2019).
3. Dong, K. *et al.* A Lithography-Free and Field-Programmable Photonic Metacanvas. *Adv. Mater.* **30,** 1703878; 10.1002/adma.201703878 (2018).
4. Shalaginov, M. Y. *et al.* Reconfigurable all-dielectric metalens with diffraction-limited performance. *Nature Communications* **12,** 1225; 10.1038/s41467-021-21440-9 (2021).
5. Karst, J. *et al.* Electrically switchable metallic polymer nanoantennas. *Science* **374,** 612–616; 10.1126/science.abj3433 (2021).
6. Galarreta, C. R. de *et al.* Nonvolatile Reconfigurable Phase-Change Metadevices for Beam Steering in the Near Infrared. *Advanced Functional Materials* **28,** 1704993; 10.1002/adfm.201704993 (2018).
7. Yin, X. *et al.* Beam switching and bifocal zoom lensing using active plasmonic metasurfaces. *Light: Science & Applications* **6,** e17016; 10.1038/lsa.2017.16 (2017).
8. Abdollahramezani, S. *et al.* Electrically driven reprogrammable phase-change metasurface reaching 80% efficiency. *Nature Communications* **13,** 1696; 10.1038/s41467-022-29374-6 (2022).
9. Ding, X. *et al.* Ultrathin pancharatnam-berry metasurface with maximal cross-polarization efficiency. *Advanced materials (Deerfield Beach, Fla.)* **27,** 1195–1200; 10.1002/adma.201405047 (2015).
10. Zhuo, W., Sun, S., He, Q. & Zhou, L. A review of high-efficiency Pancharatnam–Berry metasurfaces. *TST* **13,** 73–89; 10.1051/tst/2020133073 (2020).
11. Kang, M., Feng, T., Wang, H.-T. & Li, J. Wave front engineering from an array of thin aperture antennas. *Opt. Express* **20,** 15882–15890; 10.1364/OE.20.015882 (2012).
12. Buske, P., Völl, A., Eisebitt, M., Stollenwerk, J. & Holly, C. Advanced beam shaping for laser materials processing based on diffractive neural networks. *Opt. Express* **30,** 22798–22816; 10.1364/OE.459460 (2022).
13. Buske, P., Hofmann, O., Bonnhoff, A., Stollenwerk, J. & Holly, C. High fidelity laser beam shaping using liquid crystal on silicon spatial light modulators as diffractive neural networks. *Opt. Express* **32,** 7064–7078; 10.1364/OE.507630 (2024).
14. Goodfellow, I. *Deep learning* (MIT Press, Cambridge, Massachusetts, London, England, 2016).
15. Cui, Z. *Nanofabrication* (Springer International Publishing, 2017).

16. Stepanova, M. & Dew, S. (eds.). *Nanofabrication. Techniques and Principles* (Springer Vienna, Vienna, 2012).

17. Heßler, A., Conrads, L., Wirth, K. G., Wuttig, M. & Taubner, T. Reconfiguring Magnetic Infrared Resonances with the Plasmonic Phase-Change Material In3SbTe2. *ACS Photonics* **9,** 1821–1828; 10.1021/acsphotonics.2c00432 (2022).

18. Conrads, L. *et al.* Reconfigurable and Polarization-Dependent Grating Absorber for Large-Area Emissivity Control Based on the Plasmonic Phase-Change Material In3SbTe2. *Advanced Optical Materials* **11**; 10.1002/adom.202202696 (2023).

19. Conrads, L. *et al.* Infrared Resonance Tuning of Nanoslit Antennas with Phase-Change Materials. *ACS Nano* **17,** 25721–25730; 10.1021/acsnano.3c11121 (2023).
