# Supplementary material for: Infrared beam-shaping on demand via tailored geometric phase metasurfaces employing the plasmonic phase-change material In3SbTe2
Source: Nat Commun. 2025 Apr 18;16:3698. doi: 10.1038/s41467-025-59122-5 (PMC12008226; doi:10.1038/s41467-025-59122-5)
Supplement: Supplementary file 2 — Description of Additional Supplementary Files [file 41467_2025_59122_MOESM2_ESM.pdf]

Description of Additional Supplementary Files

Supplementary Movie 1: IST metasurface dual-hologram: Measurement and simulation of the scattered light intensity behind the dual-hologram metasurface for increasing distance.

Supplementary Movie 2: Optical programming of IST metasurfaces.
